# Pursue today and assess tomorrow - how students’ subjective perceptions influence their preference for self- and peer assessments

**DOI:** 10.1186/s12909-020-02383-z

**Published:** 2020-11-27

**Authors:** Meskuere Capan Melser, Stefan Lettner, Andjela Bäwert, Claudia Puttinger, Anita Holzinger

**Affiliations:** 1 Research Unit for Curriculum Development, Teaching Center/Medical University of Vienna, Spitalgasse 23, Bauteil 87, A-1090 Vienna, Austria; 2 University Clinic of Dentistry/Medical University of Vienna, Sensengasse 2a, Vienna, A-1090 Austria; 3 Assessment & Skills, Teaching Center/Medical University of Vienna, Spitalgasse 23, Bauteil 87, Vienna, A-1090 Austria

**Keywords:** Peer-assessment, Self-assessment, Perceptions of undergraduate medical students, Assessment for learning

## Abstract

**Background:**

Alternative assessments engage students in the assessment process to improve both short- and long-term outcomes by developing their judgments and responsibility about their own learning, and that of their peers. In this study, we investigated students’ perception towards self- and peer-assessment, their objectivity and impact on students’ learning.

**Methods:**

The study was conducted at the Medical University of Vienna. Attitudes of second year undergraduate medical students towards self- and peer-assessment, and their objectivity, appropriateness, and the impact of these assessments on students’ learning activities, was inquired using a self-developed questionnaire.

**Results:**

Four hundred twenty-three students participated in this study. Self-assessment was found more appropriate method to assess students’ knowledge. Most of students agreed that peer-assessment is not objective (M = − 0.07). Majority of students evaluated that peer assessment has no or little impact on their active and passive learning (M = − 0.23, − 0.35), on the other hand self-assessment was reported as a helpful tool for gaining long-term knowledge (M = 0.13) and following the content of courses (M = 0.16).

**Conclusion:**

Based on our results, students’ perspective on peer assessment were negative, on the whole, students had positive attitudes towards self-assessment and negative attitudes towards peer-assessment. This study also determined that self-assessment leads to the promotion of students’ learning.

## Background

Medical students not only need clinical knowledge and skills, but must also develop crucial skills such as responsibility, judgment and autonomy during their education [1]. In higher education, assessment should mainly focus on “acquisition” of learning rather than “participation” in learning [2]. However, traditional assessment methods can undermine students’ self-judgement of their own work, and lead them to become passive recipients of externally imposed assessment practices [2]. Therefore, it is important to involve students in the assessment process, which requires encouraging students to take responsibility for their own learning and enhancing development of self-observation and self-judgement [3].

Involving students in evaluation and assessment can be organised in two ways; 1) Self-assessment and 2) Peer-assessment. In self-assessment, learners use criteria and apply standards to make judgements about their achievements and the outcomes of their learning. Self-assessment is a valuable approach to supporting students’ learning and skills [4, 5], and increases the students’ future professional development and long-life learning [6]. Some researchers reported that effective feedback by self-assessment increases students learning, educational and professional standards [4, 7]. Similarly; Drew [8] found that potent feedback is critical for students to build self-confidence, and helps them evaluate themselves realistically. Thomas and his colleagues concluded that self and peer assessment improves both short- and long-term outcomes by requiring students to make sophisticated judgments about their own learning, and that of their peers. In peer-assessment, students evaluate their peers or are evaluated by their peers [9]. The goals of peer-assessment are self-directed and collaborative learning, providing detailed feedback, increasing variety and interest, identification and bonding, self-confidence, and empathy with others [10, 11].

Both, self- and peer assessment, are beneficial to students’ learning by involving them to give and receive feedback [12]. Both assessments have often been used either as summative or formative for giving the students the opportunity to gain insight about their own performance, to become self-sufficient in learning, and to think deeply and learn to constructively criticise [13, 14]. Segers and Dochy (2001) evaluated students’ perception of self and peer assessment in a problem-based learning environment setting, and reported that both assessments stimulate students’ deep-level learning and critical thinking [15]. Others compared self-assessment, peer and faculty evaluations of interviewing skills of medical students, and found that students are capable of assessing their peers but have difficulties in evaluating their own performance [16] . Additionally, dental students’ found peer and self-assessments have a positive role in the development of their reflective practical skills and appreciated the importance of reflection and learning from their peers [17]. On the other hand, they found faculty feedback more valuable than peer feedback [18]. Similarly, Sullivian et al. compared self, peer and faculty ratings in the setting of problem based tutorial groups in medical education, and pointed out that students were not able to identify their own strengths and weaknesses compared to their peers and faculty [19]. Rudy et al. investigated the reasons why students are more proficient in evaluating their peers in comparison to their own skills, knowledge and performance. Firstly, students may be socially uncomfortable in presenting a wholly favourable impression of themselves to others and prefer to be modest in their self-assessments. Another possible explanation could be a tradition of judgemental and punitive evaluation in medical education which inhibits students from expressing themselves. Alternatively students may have unrealistic goals and expectations of their abilities due to inexperience [16].

Segers and Dochy reported some concern about students’ mixed feelings about being capable of assessing each other fairly [15]. Sambell also pointed out similar concern about the reliability of self and peer assessment, even though students preferred these assessments [20]. Topping et al. also reported that friendship, sympathy or antipathy, or popularity of individuals have an effect on peer assessment [10]. These factors can influence the students’ acceptance or belief in the reliability of assessment. There is evidence that students find the grading of each other risky and unfair; they also doubt the objectivity of peer assessment, and good students seem to have a tendency to underrate their performance, whereas weaker students tend to overrate their performance [14, 21]. In contrast, students overestimate their capabilities in self-assessment in comparison to teacher-assessments [22]. Additionally, some students report that it is difficult to be objective towards oneself, and to be critical towards a peer. In both assessments, the students find it easier to assess technical aspects of the essays compared to aspects related to content skills; and all students feel that a peer’s assessment of their own essay is fair [12]. Taking these literatures into account, students’ attitudes and experiences towards self- and peer-assessment varies. These described various perceptions led us to question what our students think about these alternative assessment formats and, highlighted why it is important to understand students’ attitude or perception of self- and peer assessment. As De Grez et al. assumed, students’ perception of peer-assessment will influence the willingness to accept feedback generated by peer assessment, and this positive attitude influences students to learn most from the feedback [23]. In taking these theoretical and empirical findings forward, this study aims to understand attitudes of medical students towards self- and peer assessment in regard to its objectivity, appropriateness and relevance. Further, this study evaluated students’ view regarding the effect of peer-assessment on their learning, and students’ perception towards acceptance and seriousness of peer feedback and peer-marking. Additionally, this study was conducted to understand students’ view regarding the effects of self-assessment on their learning such as gaining long-term knowledge, following the content of the course and getting actively involved in learning activities.

## Methods

### Questionnaire

A self-created questionnaire was handed out to third semester medical and dental students (n: 432 out of 620) from December 18th to 22nd, 2017 during mandatory courses. 190 female students and 242 male students (43 dental students and 380 medical students) volunteered to fill out the anonymous questionnaire. All students signed a declaration of consent. The study was approved by the data protection and clearing committee of the Medical University of Vienna.

The study was designed as a descriptive survey. The questionnaire was based on recently used self-assessment methods including formative Medicine Progress Test (PTM) and other self-assessment tests offered by Modular Object-Oriented Dynamic Learning Environment (Moodle) and peer assessment. The questionnaire included 4 different parts that contained five to nine questions. The main focus of the survey was the objectivity, fairness and usefulness of alternative assessments, their effect on students’ learning, and their acceptance and seriousness. Along with sociodemographic information such as sex and education, the questionnaire included four different parts that addressed perceptions, opinions, and attitudes of medical/dental students towards self and peer assessment. The questionnaire measured levels of agreement on a four level Likert scale ranging from “strongly disagree” to “strongly agree”. The acceptable response rate for questionnaire was 84.9%.

### Statistical Methods

Data was entered using SPSS version 24, validity of data entry was checked by independently re-entering 10% of the data. Statistical analyses were done by using R 3.6.0.

The Likert scale of questions was first interpreted numerically from 1 to 4, and then centred so that the neutral mid-point of the scale is zero. Afterwards, the arithmetic mean, standard deviations, as well as a 95% confidence interval for the mean were calculated by using the bootstrap [24], and presented graphically as error bars.

## Results

**Fig. 1:**
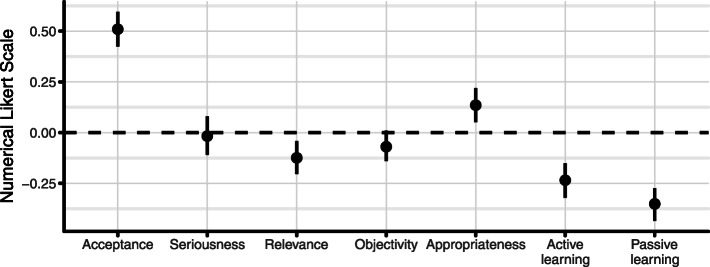
Students’ view about Peer Assessment

**Fig. 2:**
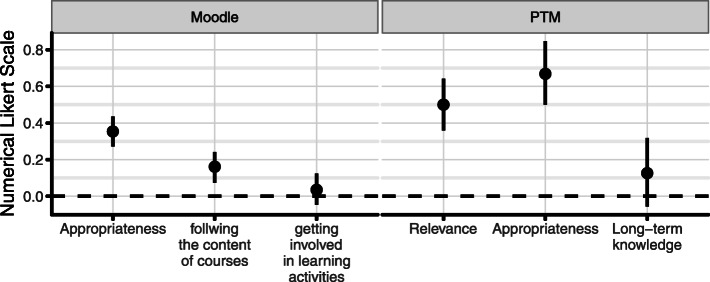
Students’ view about Self-Assessment

Majority of students found peer assessment not objective, with a mean of − 0.07 (SD = 0.80, CI [− 0.14 to- 0.01]) (Fig. 1). Most students reported that the content of the peer assessment is not relevant (M = − 0.12, SD = 0.87, CI [− 0.20 to − 0.04] for them (Fig. 1). On the other hand, requested content of self-assessment was found relevant (M = 0.50, SD = 0.70, CI [0.36 to 0.64]) (Fig. 2).

Most of students considered peer feedback as acceptable as that of teachers (M = 0.51, SD = 0.88, CI [0.42 to 0.59]), but they also reported that they took peer-marking less seriously (M = − 0.02, SD = 0.97, CI [− 0.11 to 0.08]) (Fig. 1). Students also considered that self-assessments are a more appropriate method to assess their knowledge (M = 0.67, SD = 0.85, CI [0.50 to 0.85]) than peer-assessment (Figs. 1 and 2). Peer-assessment was considered less appropriate (M = 0.14, SD = 0.90, CI [0.05 to 0.22**])** (Fig. 1).

Students considered that peer assessment has little or no effect on their learning (active: M = − 0.23, SD = 0.88, CI [− 0.32 to − 0.15]; passive: M = − 0.35, SD = 0.87, CI [− 0.44 to − 0.27]) (Fig. 1). In comparison, students reported that self-assessment leads them to gain long-term knowledge (M = 0.13, SD = 0.91, CI [− 0.06 to 0.32]) (Fig. 2). Students also reported that self-assessments definitely help them to follow the content of courses (M = 0.16, SD = 0.92, CI [0.07 to 0.24]) (Fig. 2) and to be better involved in learning activities (M = 0.04, SD = 0.94, CI [− 0.05 to 0.12]) in comparison to peer-assessment (Figs. 1 and 2).

## Discussion

In this study, alternative assessment formats were explored. Students’ perception towards peer- and self-assessment methods and the impact of peers’ feedback on students’ learning activity were evaluated. Students’ view about objectivity of peer assessment and acceptance and seriousness of peer feedback and peer-marking were also questioned.

With regard to the research question focusing on student’s perceptions of self and peer assessment, our results reflected a positive attitude towards the value of self-assessment. Students found that self-assessment affects the outcomes of their learning positively. Regarding students’ perceptions of peer assessments, students found peer feedback acceptable, however the majority reported not to take peer-marking seriously. Peer assessment was also considered not objective by students.

Assessments are one of the crucial component in the process of teaching and learning. The advantages of self- and peer-assessment have been reported by several researchers. According to Zimmerman and Schunk, self-assessment involves a wide range activities of self-regulation which is a core competence required for learning to learn [25]. Self-assessment plays an important role in self-regulated learning and can significantly increase the amount of knowledge students can gain from self-regulated learning, such as self-reflection, self-observation, and self-motivation, in which they choose their own learning tasks [26]. In peer assessment, students are directly engaged in training self-monitoring, self-evaluation and task-selection skills, in all of which the students have much control over the learning tasks they are engaged in. Researchers also revealed that the self-regulation of the learners who practice both self- and peer-assessment practices improve significantly [27]. Beside these advantages, students felt that self- and peer-assessment encouraged them to compare and reflect on their own work; these methods gave them the opportunity to develop collaborative skills, engagement in reflection and exploration of ideas, and enable students to work together in the sense of developing collaborative skills, practicing, planning and teamwork and of [2, 28].

On the other hand, students’ ability to assess each other may influence the objectivity of peer assessment. Ballantyne et al. reported that the things students disliked about peer assessment included: questioning peers competency in marking, issues of fairness, and objectivity [28]. White also addressed that friendship can play an important role in the objectivity of peer assessment, students may give good scores to close friends and bad scores to others. The fear of peers’ misunderstanding and the possibility of being discriminated against could affect the objectivity of peer assessment [29]. Similar to White, in this study, we found students’ negative attitudes towards objectivity of peer assessment. As Yunella [30] concluded, the objectivity of peer assessment can be achieved if students get clear instructions from their teacher. Therefore, teacher intervention about the objectivity of peer assessment plays a crucial role in applying peer assessment [30]. In contrast, investigations showed that peer assessment can be a relevant substitute for assessment by a teacher and, can be as objective as teacher assessment [31].

Another disadvantage of peer assessment is that students may not take peer feedback seriously. Our study showed that students found peer feedback acceptable in a positive way, however they do not take peer-marking seriously. The reasons for this could be firstly that students may think that assessing and marking are teacher’s job, and that the teacher is a more experienced assessor, and have more competence in assessment criteria. Secondly that students may not be serious in assessing their peers because of hierarchical thinking, and they may see a teacher as a person with a higher hierarchy than peers. Similarly, the perception that assessment by a teacher is more reliable and more valid than the assessment by the peer may lead students to regard peer feedback, as well as peer marking, not seriously. Furthermore self-assessed grades tend to be higher than staff grades [32]. To minimize these concerns, some researchers offered following suggestions: 1) the application of specific criteria [33]; 2) transparency in assessment process [7, 34]; 3) good instructions and training of students’ assessment skills [35]; and 4) the use of scoring matrix [36]. Lindblom-Ylänne and her colleagues (2006) investigated whether the use of matrix enhanced the accuracy of self and peer assessment of essays, and found that specific criteria such as critical thinking, use of literature, appearance, length etc., and good instructions for students seemed to enhance the accuracy. They also explored how students and teachers perceive their experiences in relation to these assessments. They reported that teachers found the use of both matrix and self- and peer-assessment in addition to teacher’s assessment of student essays was very positive [12]. Other researchers pointed out that there was no significant difference in the overall mark averages given by peers and that given by their teachers, and concluded that peer assessment can be as objective as teacher assessment [23, 37–39].

Self- and peer-assessment can enhance student learning, gain more work-related skills and work-integrated learning [40, 41], and develop taking responsibility for their own learning; a better understanding of the subject matter, and their own values and judgements and critical reflection skills [6, 42, 43]. On the other hand, our students found no impact of peer-assessment on both their active and passive learning. The reason could be students’ negative perceptions of the objectivity of peer-assessment. Researchers have also shown that issues of bias, trust and capability play on students’ minds during self and peer-assessment activities [44]. According to Segers and Dochy (2001) students generally found that the process of assessing themselves stimulates their deep-level learning and critical thinking [15].

Similarly, our students believe that self-assessment leads them to gain long-term knowledge as well as that self-assessment enables them to participate actively in assessment and learning activities.

Taken together, our findings indicate that students do have positive attitudes towards self-assessment but negative perceptions towards peer-assessment, because of the lack of objectivity of peer-assessment, and questioning the seriousness of peer feedback and peer-marking. In addition, our study provides evidence that self-assessment leads students to stimulate deep-level learning, and to prepare themselves better for assessment and learning activities. There is still a growing need for literature about student perceptions of self and peer-assessment. Most of the existing studies only aim to evaluate the design and implementation of self and peer-assessment [35, 45]. Nicol, Thomson, and Breslin (2014) stated that ‘there is no doubt that more research is required on peer review and its different components, including more studies of students’ experiences, perceptions and responses to the different feedback arrangements that are possible during its implementation’ [46].

In conclusion, this study shows that students have a certain justification for both assessments. Students see the positive benefits of self- and peer assessment and positive impact of these assessments on their learning, but they are also aware of the potential disadvantages of these assessments. Therefore, it is important to consider the above mentioned approaches such as transparency, scoring matrix, introduction and training for students and application criteria for implementing these assessments properly into a curriculum.

## Limitations

Overall, the study has provided valuable information. Study limitations include the opinion of students who were in second year of undergraduate medical education and not yet more experienced in one of the self-assessments (formative Medicine Progress Test (PTM)) in comparison to peer-assessment and other self-assessment tests offered by Modular Object-Oriented Dynamic Learning Environment (Moodle).

## Data Availability

The questionnaire used during this study is available from the corresponding author on reasonable request.
